# Association between length of storage of red blood cell units and outcome of critically ill children: a prospective observational study

**DOI:** 10.1186/cc8953

**Published:** 2010-04-08

**Authors:** Oliver Karam, Marisa Tucci, Scot T Bateman, Thierry Ducruet, Philip C Spinella, Adrienne G Randolph, Jacques Lacroix

**Affiliations:** 1 Pediatric Critical Care Unit, CHU Sainte-Justine, Université de Montréal, 3175 chemin de la Côte Sainte-Catherine, Montreal H3T 1C5, Canada; 2 Department of Pediatrics, University of Massachusetts Medical Center, 55 Lake Avenue, North Worcester, MA 01655, USA; 3 Department of Pediatrics, Connecticut Children's Medical Center, 282 Washington St, Hartford, CT 06106, USA; 4 Department of Surgery, Connecticut Children's Medical Center, 282 Washington St, Hartford, CT 06106, USA; 5 Division of Critical Care Medicine, Department of Anesthesia, Perioperative and Pain Medicine, Children's Hospital, 300 Longwood Avenue, Boston, Massachusetts 02115, USA

## Abstract

**Introduction:**

Transfusion is a common treatment in pediatric intensive care units (PICUs). Studies in adults suggest that prolonged storage of red blood cell units is associated with worse clinical outcome. No prospective study has been conducted in children. Our objectives were to assess the clinical impact of the length of storage of red blood cell units on clinical outcome of critically ill children.

**Methods:**

Prospective, observational study conducted in 30 North American centers, in consecutive patients aged <18 years with a stay ≥ 48 hours in a PICU. The primary outcome measure was the incidence of multiple organ dysfunction syndrome after transfusion. The secondary outcomes were 28-day mortality and PICU length of stay. Odds ratios were adjusted for gender, age, number of organ dysfunctions at admission, total number of transfusions, and total dose of transfusion, using a multiple logistic regression model.

**Results:**

The median length of storage was 14 days in 296 patients with documented length of storage. For patients receiving blood stored ≥ 14 days, the adjusted odds ratio for an increased incidence of multiple organ dysfunction syndrome was 1.87 (95% CI 1.04;3.27, *P *= 0.03). There was also a significant difference in the total PICU length of stay (adjusted median difference +3.7 days, *P *< 0.001) and no significant change in mortality.

**Conclusions:**

In critically ill children, transfusion of red blood cell units stored for ≥ 14 days is independently associated with an increased occurrence of multiple organ dysfunction syndrome and prolonged PICU stay.

## Introduction

Almost half of all critically ill patients, adults as well as children, admitted to a critical care unit for more than 48 hours will receive a red blood cell (RBC) transfusion during their stay [1,2]. RBC transfusions constitute a potentially life-saving intervention aimed at restoring hemoglobin levels, to maintain adequate oxygen delivery to vital organs. However, some data suggest that they can also put critically ill patients at risk for significant complications including increased rates of mortality [3,4], increased multiple organ dysfunction syndrome (MODS) [2,5-7], acute respiratory distress syndrome (ARDS) [8], deep vein thrombosis [9] and nosocomial infections [10-14]. Storage of RBC units is essential, because it allows the separation in time and space of donation and transfusion and it improves the availability of blood products. Presently, the maximum recommended length of storage, which is based on a 24-hour post-infusion *in vivo *recovery of more than 75% of RBC, is 42 days with the preservative solutions currently used in Canada and the USA [15-18].

Blood banks do not issue blood in a random order: the standard practice is to dispense the oldest blood available in order to reduce potential waste. In recent years, several studies have addressed the issue of RBC unit length of storage and its clinical effects in adults who require transfusions. Whereas some have reported a worse clinical outcome in patients transfused with older blood [6,19-21], others did not find any association between RBC length of storage and increased morbidity or mortality [22-25]. Differences in these conflicting studies, which include baseline severity of illness of patients studied and sample size issues, make comparing these studies difficult. Only one small retrospective study has assessed the effect of RBC length of storage on outcomes in children and no relation was found between RBC unit length of storage and clinical outcome in critically ill children [26].

The primary objective of this observational study was to assess the relation between RBC length of storage and the development of new or progressive MODS in critically ill children, by analyzing data from a large prospective pediatric intensive care unit (PICU) transfusion study [2]. Secondary objectives included the evaluation of the relation between RBC length of storage and adverse outcome as reflected by mortality and PICU length of stay.

We report an independent association between transfusion of RBC units with more prolonged storage time and a higher occurrence rate of new or progressive MODS in critically ill children.

## Materials and methods

This study involves patients recruited in a prospective, epidemiological, observational study conducted in 30 PICUs by the Pediatric Acute Lung Injury and Sepsis Investigators (PALISI) Network in the USA and Canada from September 2004 to March 2005 [2]. All children aged less than 18 years who were admitted to a participating PICU and whose length of stay was more than 48 hours were eligible. Institutional review board approval was obtained at all study sites. Written informed consent was obtained for all enrolled subjects.

Some data from the first 48 hours after PICU admission were collected retrospectively, and the rest of the data were collected prospectively up to a maximum of 28 days in the PICU, or until hospital discharge, inter-institutional transfer or death. Any patient readmitted within 48 hours of PICU discharge was attributed only one ICU stay.

Data collected on admission included: demographic data, severity of illness as estimated by the Pediatric Risk of Mortality (PRISM) III score [27], organ dysfunction as estimated by the Pediatric Logistic Organ Dysfunction (PELOD) score [28] and the MODS score [29]. Daily data collection included RBC transfusion events, length of storage of RBC units, MODS variables, clinical information and complications.

The total number of transfusions was recorded for each patient, as well as the volume transfused per transfusion. The total dose of transfusion standardized for body weight was computed by dividing the total volume administered by the patient weight at PICU admission.

RBC concentrates stored for a period shorter than the median length of storage were defined as 'fresh blood', whereas those stored for more than the median length of storage were defined as 'old blood'. For patients requiring multiple transfusions, 'old blood' or 'fresh blood' attribution was based on the oldest unit received. To compute the median length of storage, the longest length of storage was used for patients receiving multiple transfusions.

The primary outcome measure was the proportion of patients who developed concurrent dysfunction of two or more organ systems (defined as MODS [30]), or had progression of MODS, as evidenced by the worsening of one or more organ dysfunctions, as described by Proulx and colleagues [30]. The secondary outcomes analyzed were PICU length of stay and 28-day mortality. All primary and secondary outcomes were monitored prospectively and were checked for after the first transfusion.

Chi-squared tests and Fisher's exact probability tests were used to undertake unadjusted bivariate tests in order to establish an association between the outcomes and categorical variables. For continuous variables, Student t tests were used. Correlations between two continuous variables were analyzed with Pearson's correlation test. Logistic regression was used to compare odds ratios for development of the primary outcome and adjustments were made for variables associated with the primary outcome: gender, age, MODS score at admission, mechanical ventilation at admission, total number of transfusions and total dose of transfusion. We also tested for an interaction between number of transfusions and total dose of transfusions. A Cox regression model, using the same covariables, was used to analyze the adjusted PICU length of stay and the time between the first transfusion and development of the primary outcome. All statistical analyzes were performed with SPSS version 16 for Mac (SPSS, Chicago, IL, USA).

## Results

### Population

A total of 977 consecutively admitted patients were enrolled in 30 sites. One center (47 patients) was excluded from analysis *a posteriori *because that center did not record the RBC unit length of storage. In the remaining 930 patients, 447 (49%) were transfused and received a total of 1991 transfusions: 176 patients (39%) were only transfused once and 271 (61%) had multiple transfusions. Eighty-six percent (86%) of the transfusions were pre-storage leukoreduced.

Data on the length of storage were available for 296 of 447 (66%) transfused patients. The proportion of missing data was not related to the participating center (*P *= 0.65). Of the 296 patients analyzed, 98 (33%) patients received only one transfusion while 198 (67%) received multiple transfusions.

### Demographic data

**Figure 1:**
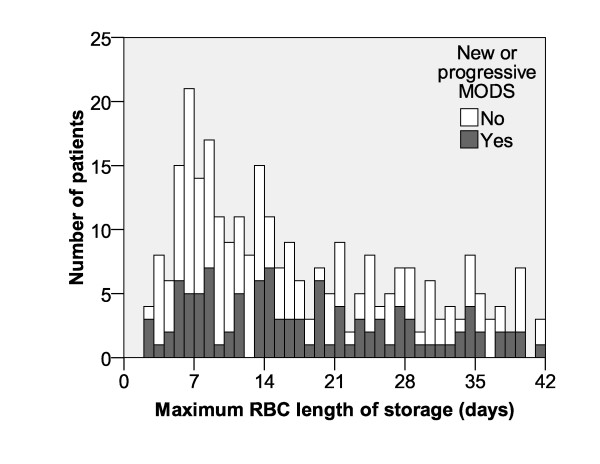
**Distribution of RBC length of storage**. The horizontal axis represents the red blood cell (RBC) length of storage (in days). The vertical axis represents the number of patients who received transfusions for each known length of storage. The black part of each bar of the histogram represents the number of patients who developed new or progressive multiple organ dysfunction score (MODS). For patients receiving multiple transfusions, the longest length of storage was used. The median length of storage is 14 days, and the mean length of storage is 17.2 days.

**Figure 2:**
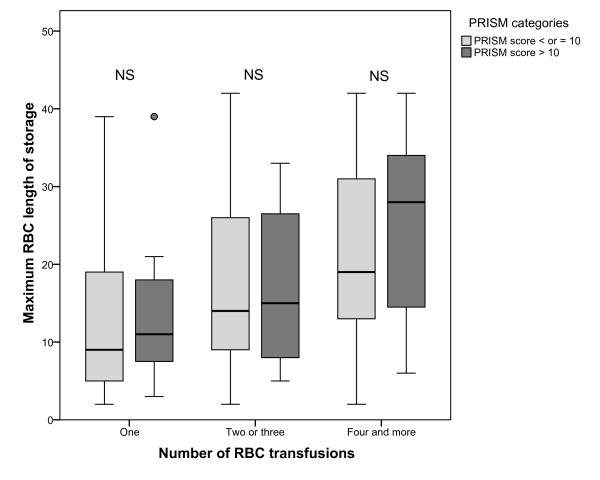
**Box plot of the maximum RBC length of storage, according to the number of RBC transfusions and according to the severity of disease at admission (PRISM III score ≤ 10 versus >10)**. RBC, red blood cell; PRISM, pediatric risk of mortality; NS, not significant.

Demographic data for transfused patients for whom length of storage data was documented are shown in Table 1. The median length of storage was 14.0 days and the mean length of storage was 17.8 ± 11.6 days (Figure 1). Infants less than one month old had a higher probability of receiving RBC units stored for less than 14 days (61% vs. 43%, *P *< 0.001). The median RBC unit length of storage was significantly higher in patients who received more than one transfusion (R = 0.24, *P *< 0.001); this correlation did not change significantly with severity of illness (Figure 2). There were no significant differences when comparing the demographic data and severity of illness at admission of patients for whom at least one RBC length of storage was documented (n = 296) and those for whom no length of storage was recorded (n = 151).

**Table 1:** Demographic data in transfused patients with documented RBC length of storage

	Transfused patients (n = 296)
Age (months), mean ± SD	58.7 ± 69.2
Gender (male), mean ± SD	171 (57.7%)
Weight (kg), mean ± SD	21.0 ± 23.0
Race	
White, n (%)	215 (72.6%)
Black, n (%)	38 (12.8%)
Asian, n (%)	8 (2.7%)
Other, n (%)	35 (11.8%)
Country	
USA, n (%)	260 (87.8%)
Canada, n (%)	36 (12.2%)
Reason of admission	
Cardiovascular, n (%)	106 (35.8%)
Respiratory, n (%)	81 (27.4%)
Central nervous system, n (%)	43 (14.5%)
Other, n (%)	66 (22.3%)
Sepsis at admission, n (%)	29 (14.8%)
Mechanical ventilation at admission	156 (52.7%)
PRISM III score at admission, mean ± SD	5.5 ± 5.7
PELOD score at admission, mean ± SD	12.0 ± 9.8
MODS at admission, mean ± SD	1.5 ± 1.2

MODS, multiple organ dysfunction score; PELOD, pediatric logistic organ dysfunction; PRISM, pediatric risk of mortality; RBC, red blood cell; SD, standard deviation.

Forty-nine percent of the transfused patients received their first transfusion within the first day after PICU admission; an additional 19% were transfused within 48 hours.

### Primary outcome

New or progressive MODS was associated with the following confounding variables at admission (Table 2): gender (odds ratio female/male 0.53, 95% confidence interval (CI) = 0.33 to 0.85, *P *= 0.01), severity of illness (MODS score mean difference 0.89, 95% CI = 0.68 to 1.10, *P *< 0.001) and mechanical ventilation (odds ratio for being ventilated 0.50, 95% CI = 0.31 to 0.80, *P *= 0.004). In patients who developed new or progressive MODS compared with those who did not, we found that the number of RBC transfusions was significantly higher (5.5 ± 5.7 vs. 2.6 ± 3.6, *P *< 0.001, respectively), the total volume of RBC transfusions was higher (72 ± 114 vs. 44 ± 79 ml/kg, *P *< 0.001, respectively), and the proportion of patients who received at least one RBC unit stored for 14 days or longer was greater (62.3% vs. 47.3%, *P *= 0.01, respectively).

**Table 2:** Confounding variables at admission according to occurrence of new or progressive MODS

	Absence of new or progressive MODS (n = 182)	Presence of new or progressive MODS (n = 114)	*P *value
Age (months), mean ± SD	59.8 ± 68.9	57.0 ± 69.9	0.73
Gender (Male), n (%)	116 (63.7%)	55 (48.2%)	0.01
Weight (kg), mean ± SD	22.1 ± 22.9	19.2 ± 23.2	0.29
PRISM III at admission, mean ± SD	6.1 ± 6.3	4.4 ± 4.4	0.008
MODS at admission, mean ± SD	1.8 ± 1.2	1.0 ± 1.0	<0.001
PELOD at admission, mean ± SD	13.1 ± 9.7	10.3 ± 9.7	0.02
Sepsis at admission, mean ± SD	22 (12.1%)	7 (6.1%)	0.11
Mechanical ventilation at admission, mean ± SD	108 (59.3%)	48 (42.1%)	0.004

MODS, multiple organ dysfunction score; PELOD, pediatric logistic organ dysfunction; PRISM, pediatric risk of mortality; SD, standard deviation.

The unadjusted odds ratio for development of new or progressive MODS in patients receiving at least one RBC unit stored for 14 days or longer was 1.84 (95% CI = 1.14 to 2.97, *P *= 0.01; Table 3). The following organs contributed to the observed MODS: 80 (27%) gastro-intestinal dysfunction, 51 (17%) cardiovascular dysfunction, 30 (10%) respiratory dysfunction, 21 (7%) hematological dysfunction, 19 (6%) renal dysfunction, and 2 (1%) neurological dysfunction. The only organ failure that differed significantly depending on RBC length of storage was renal failure (*P *= 0.02).

**Table 3:** Demographic, transfusion related and outcome variables according to length of RBC storage

	**RBC unit length of storage** **<14 days** **(n = 139)**		***P *value** **≥ 14 days** **(n = 157)**
Age (months), mean ± SD	37.5 ± 56.8	77.4 ± 73.8	<0.001
Male, n (%)	87 (62.6%)	84 (53.5%)	0.12
Weight (kg), mean ± SD	15.3 ± 18.5	26.0 ± 25.4	<0.001
PRISM III at admission, mean ± SD	4.9 ± 5.7	6.0 ± 5.7	0.09
MODS at admission, mean ± SD	1.3 ± 1.2	1.6 ± 1.3	0.04
PELOD at admission, mean ± SD	10.0 ± 8.3	13.8 ± 10.8	0.001
Sepsis at admission, n (%)	13 (9.4%)	16 (10.2%)	0.85
Mechanical ventilation at admission, n (%)	77 (55.4%)	83 (52.9%)	1.00
Total number of RBC transfusions, mean ± SD	2.6 ± 3.6	5.5 ± 5.7	<0.001
Total dose of RBC transfusions (ml/kg), mean ± SD	44 ± 79	72 ± 114	0.02
New or progressive MODS, n (%)	43 (30.9%)	71 (49.3%)	0.01
Death at 28 days, n (%)	6 (4.3%)	9 (6.3%)	0.61
PICU length of stay (days), mean ± SD	9.9 ± 8.3	14.0 ± 10.4	<0.001

Results are expressed as mean ± standard deviation or numbers and proportions.

MODS, multiple organ dysfunction score; PELOD, pediatric logistic organ dysfunction; PICU, pediatric intensive care unit; PRISM, pediatric risk of mortality; RBC, red blood cell; SD, standard deviation.

After correction for confounding variables (gender, age, MODS at admission, mechanical ventilation at admission, total number of transfusions and total volume of transfusion), the adjusted odds ratio for development of new or progressive MODS in patients receiving older blood (stored ≥ 14 days) was 1.87 (95% CI = 1.04 to 3.27, *P *= 0.03). The Hosmer-Lemeshow goodness-of-fit test for this model was 0.49.

In patients who received a single transfusion with a documented length of storage (n = 98), the adjusted odds ratio for development of new or progressive MODS was 2.36 (95% CI = 0.88 to 6.34, *P *= 0.09) in those receiving a RBC unit stored for 14 days or longer.

Patients also had an independently greater risk of developing new or progressive MODS, which increased by a factor of 1.13 (95% CI = 1.03 to 1.24, *P *= 0.01) for each RBC transfusion.

### Secondary outcomes

In the univariate analysis, the mean PICU length of stay was significantly longer for patients receiving old blood (stored ≥ 14 days) compared with those receiving fresh blood (9.9 ± 8.3 days vs. 14.0 ± 10.4 days, mean difference 4.1 days, 95% CI = 2.0 to 6.3, *P *< 0.001; Table 3). There was no significant difference for mortality (6.3% vs. 4.3%, *P *= 0.6).

**Figure 3:**
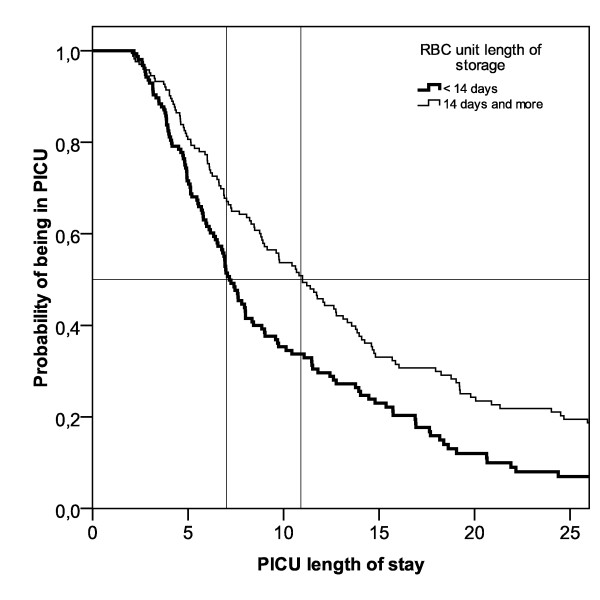
**Adjusted PICU length of stay, according to RBC unit length of storage**. The Cox regression model is adjusted for gender, age, multiple organ dysfunction score (MODS) at admission, mechanical ventilation at admission, total number of transfusions, and total transfusion dose. Adjusted median difference in pediatric intensive care unit (PICU) length of stay was 3.7 days (*P *< 0.001); hazard ratio = 1.39 (95% CI = 1.07 to 1.80, *P *= 0.01).

Using the logistic models, there was also a significant difference in the adjusted median length of PICU stay (adjusted median difference +3.7 days, *P *< 0.001; hazard ratio 1.39, 95% CI = 1.07 to 1.80, *P *= 0.01) for patients receiving old blood (Figure 3), but no significant impact on mortality.

**Figure 4:**
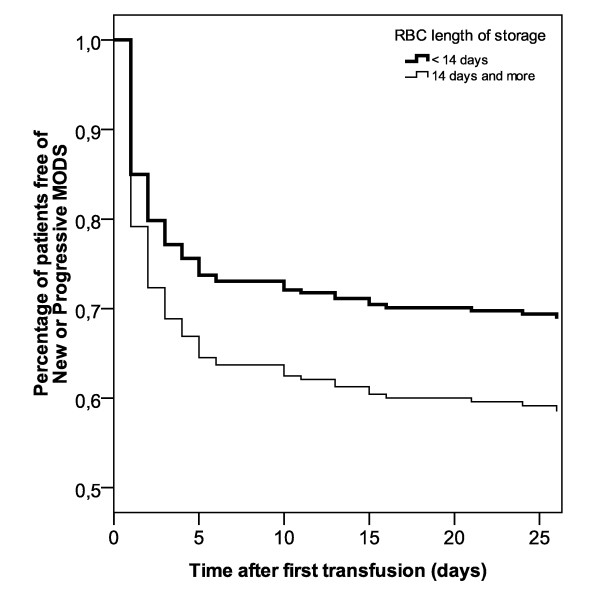
**Time to develop new or progressive MODS**. Adjusted proportion of patients free of primary outcome (new or progressive multiple organ dysfunction score (MODS)), according to the red blood cell (RBC) length of storage (<14 days versus ≥ 14 days). The Cox regression model was adjusted for gender, age, MODS at admission, mechanical ventilation at admission, total number of transfusions, and total transfusion volume. Hazard ratio = 1.43 (95% CI = 0.96 to 2.15, *P *= 0.08).

We evaluated the time between the first transfusion and the occurrence of new or progressive MODS (Figure 4). Patients who received older blood had a trend toward developing new or progressive MODS faster than those who received fresh blood (hazard ratio = 1.43, 95% CI = 0.96 to 2.15, *P *= 0.08).

## Discussion

This observational study evaluates the clinical impact of RBC unit length of storage in critically ill children. We report an independent association between more prolonged RBC unit length of storage and increased morbidity: patients who are transfused with at least one RBC unit stored for 14 days or longer had a significantly higher risk of new or progressive MODS and a longer PICU length of stay.

The relation between RBC unit length of storage and clinical outcome has been extensively debated recently. The results of many large observational studies in adults are conflicting. Some authors reported that transfusion of older RBC units (generally a storage time >14 days) is associated with adverse events that include diminished cerebral oxygenation [31], increased rate of nosocomial infections [19], increased deep vein thrombosis [9], increased MODS [6], and increased mortality [3,9,20,21]. Others reported no significant clinical impact with transfusion of older RBC units [22-25,32]. The only pediatric study evaluating the effect of RBC unit length of storage on outcome was a *post-hoc *analysis by Kneyber and colleagues [26]. They reported no differences in length of ventilation, PICU length of stay, or death rate in a small number of transfused patients (n = 67). Our data show that RBC units stored for 14 days or longer are independently associated with a worse clinical outcome, as reflected by the occurrence of new or progressive MODS and by the PICU length of stay.

Several possible mechanisms may explain the adverse clinical effects that are reported with transfusion of older RBC units. Various biochemical changes occur during the storage process, such as a decrease in 2,3-diphosphoglycerate and S-nitrosohemoglobin, which regulates the vasodilatory response to local hypoxemia [33,34]. This could result in an increased mismatch that may compromise oxygen supply to certain tissues. This has been recently observed clinically by Kiraly and colleagues, who reported a decreased tissue oxygenation in patients receiving older blood transfusions [35]. Older RBCs are less deformable [36], contain more extracellular ubiquitin [37] and advanced glycation end-products [38], express more phosphatidylserine [39], and induce more cytokine production [40] and secretory phospholipase A2 [41]. All these changes in stored RBCs are known to have immunologic or pro-coagulant properties, which could possibly increase the risk of poor outcomes, including multiple organ failure.

Our data also show an independent association between the number of RBC transfusions and the occurrence of new or progressive MODS, every additional transfusion increasing the odds of developing this outcome by 13%. Such a relation has also been described by others [4,42,43]. A higher number of transfusions exposes the patient to more antigens and more inflammatory mediators, which may alter his immune status. In addition, patients with multiple transfusions have a higher mathematical probability of receiving at least one older RBC unit. A relation between severity of illness at baseline and multiple transfusions is also frequently reported. The data reported in the medical literature showed repeatedly a strong association between older RBC units, severity of illness, and/or more RBC transfusions, and worse outcome in critically ill patients, but it is almost impossible to determine if it is the length of storage, the number of transfusions, or the severity of illness that explained worse outcome. Our study shows that worse clinical outcome is associated with the number of transfusions independently of the longest length of storage; such an independent relation has only been reported recently in adult trauma patients [9,21]. This implies that all studies assessing the association between length of storage and clinical outcome must take into account not only the age of the blood products, but also the total number of transfusions administered and the severity of illness.

There are several limitations in our study. The main limitation is that RBC unit length of storage was not available for one-third of the patients. Although it was not possible to recuperate the missing data, we do not anticipate that the cohort of patients with missing data would bias the results because missing data were not related to the severity of illness at admission. Furthermore, these missing data did not allow us to analyze the data according to other RBC length of storage cutoffs due to sample size issues. However, further support that our findings are valid comes from our analysis of the subgroup of patients who received only one transfusion whose length of storage was available and unequivocal. Although we did not attain sufficient statistical power, there was a trend for a higher adjusted odds ratio for developing new or progressive MODS (2.36, *P *= 0.09, n = 98) in those who received blood older than 14 days.

There are other limitations. It has been suggested that leukoreduction is associated with a better clinical outcome [44]. Although it would have been ideal to include this covariable in our logistic regression, the database did not provide sufficient data on leukoreduction to allow for this adjustment. However, because most transfusions (86%) were leukoreduced, there is not sufficient power to analyze this variable. Infants got fresher blood than older children. This might be due to blood bank policies whereby fresher blood may have been provided for cardiac surgery patients, who are likely to be younger. However, our logistic models adjusted for patient age. In patients who received multiple transfusions, analysis was subject to confounding influences due to the mixture of storage times. Although it seems reasonable to adjudicate to the 'older blood' group those who had received at least one transfusion of old blood, one could argue that the groups should be allocated according to the freshest blood administered, or according to the mean or the median length of storage, or perhaps according to a weighted average of the length of storage all RBC units received. The best way to address length of storage issues in patients who received multiple transfusions remains to be determined. Despite the use of maximum RBC age to define old RBCs, which biases our results towards the null hypothesis, our analysis indicated a significant independent association between RBC unit length of storage and both the occurrence of new or progressive MODS and a more prolonged PICU length of stay. Caution is warranted in the interpretation of these results, which show an association between RBC length of storage and a more adverse clinical outcome in critically ill children. We must underline the fact that our study reported an independent association, not a cause-effect relation between more prolonged length of storage of RBC units and outcome in critically ill patients: only a randomized clinical trial on this question may prove that such cause-effect relation is real.

## Conclusions

This observational pediatric study suggests that critically ill children receiving RBC units stored for 14 days or longer are at greater risk of developing new or progressive MODS. Despite the limitations of our study, the observation of an independent association between longer length of storage and a greater risk of new or progressive MODS in critically ill children is a novel and important finding. Definitive conclusions cannot be drawn, but these observational data justify undertaking a randomized controlled trial to evaluate the effect of RBC length of storage in critically ill children.

## Key messages

• The clinical impact of the transfusion of RBC units with a more prolonged storage time is a controversial issue. Conflicting results on morbidity and mortality have been published in adults. No large prospective studies have addressed this question in critically ill children.

• In this study, we prospectively evaluate the association between prolonged RBC storage time and clinical outcome in critically ill children.

• In critically ill children, transfusion of RBC units stored for 14 days or longer is independently associated with an increased occurrence of MODS and prolonged PICU stay.

• These novel and important observational data justify undertaking a randomized controlled trial to evaluate the effect of RBC length of storage on the outcome of critically ill children.

## Abbreviations

ARDS: acute respiratory distress syndrome; CI: confidence interval; MODS: multiple organ dysfunction syndrome; PALISI: pediatric acute lung injury and sepsis investigators; PELOD: pediatric logistic organ dysfunction; PICU: pediatric intensive care unit; PRISM: pediatric risk of mortality; RBC: red blood cell.

## Competing interests

The authors declare that they have no competing interests.

## Authors' contributions

OK participated in the design of the study and drafted the manuscript. MT and PCS participated in the design of the study and helped to draft the manuscript. TD performed the statistical analysis and helped to draft the manuscript. SB, AGR and JL conceived of the study and helped to draft the manuscript. All authors read and approved the final manuscript.

## References

[B1] Corwin HL Gettinger A Pearl RG Fink MP Levy MM Abraham E MacIntyre NR Shabot MM Duh MS Shapiro MJ The CRIT Study: Anemia and blood transfusion in the critically ill--current clinical practice in the United States Crit Care Med 2004 32 39 52 10.1097/01.CCM.0000104112.34142.79 14707558

[B2] Bateman S Lacroix J Boven K Forbes P Barton R Thomas N Jacobs B Markovitz B Goldstein B Hanson J Li H Randolph A Anemia, blood loss, and blood transfusions in North American children in the intensive care unit Am J Resp Crit Care Med 2008 178 26 33 10.1164/rccm.200711-1637OC 18420962

[B3] Spinella PC Perkins JG Grathwohl KW Beekley AC Niles SE McLaughlin DF Wade CE Holcomb JB Effect of plasma and red blood cell transfusions on survival in patients with combat related traumatic injuries J Trauma 2008 64 S69 77 discussion S77-68. 10.1097/TA.0b013e318160ba2f 18376175

[B4] Kneyber MC Hersi M Twisk JW Markhorst D Plötz F Red blood cell transfusion in critically ill children is independently associated with increased mortality Intensive Care Med 2007 33 1414 1422 10.1007/s00134-007-0741-9 17572875

[B5] Sauaia A Moore FA Moore EE Haenel JB Read RA Lezotte DC Early predictors of postinjury multiple organ failure Arch Surg 1994 129 39 45 8279939 10.1001/archsurg.1994.01420250051006

[B6] Zallen G Offner PJ Moore EE Blackwell J Ciesla DJ Gabriel J Denny C Silliman CC Age of transfused blood is an independent risk factor for postinjury multiple organ failure Am J Surg 1999 178 570 572 10.1016/S0002-9610(99)00239-1 10670874

[B7] Aiboshi J Moore EE Ciesla DJ Silliman CC Blood transfusion and the two-insult model of post-injury multiple organ failure Shock 2001 15 302 306 10.1097/00024382-200115040-00009 11303730

[B8] Gong M Thompson BT Williams P Pothier L Boyce P Christiani D Clinical predictors of and mortality in acute respiratory distress syndrome: potential role of red cell transfusion* Crit Care Med 2005 33 1191 1198 10.1097/01.CCM.0000165566.82925.14 15942330

[B9] Spinella PC Carroll CL Staff I Gross R Mc Quay J Keibel L Wade CE Holcomb JB Duration of red blood cell storage is associated with increased incidence of deep vein thrombosis and in-hospital mortality in patients with traumatic injuries Crit Care 2009 13 R151 10.1186/cc8050 19772604 PMC2784373

[B10] Hill GE Frawley WH Griffith KE Forestner JE Minei JP Allogeneic blood transfusion increases the risk of postoperative bacterial infection: a meta-analysis J Trauma 2003 54 908 914 10.1097/01.TA.0000022460.21283.53 12777903

[B11] Vamvakas EC Blajchman MA Universal WBC reduction: the case for and against Transfusion 2001 41 691 712 10.1046/j.1537-2995.2001.41050691.x 11346708

[B12] Shorr AF Jackson WL Transfusion practice and nosocomial infection: assessing the evidence Curr Opin Crit Care 2005 11 468 472 10.1097/01.ccx.0000176689.18433.f4 16175034

[B13] Vamvakas EC Blajchman MA Transfusion-related immunomodulation (TRIM): an update Blood Rev 2007 21 327 348 10.1016/j.blre.2007.07.003 17804128

[B14] Gunst MA Minei JP Transfusion of blood products and nosocomial infection in surgical patients Curr Opin Crit Care 2007 13 428 432 10.1097/MCC.0b013e32826385ef 17599014

[B15] Simon TL Marcus CS Myhre BA Nelson EJ Effects of AS-3 nutrient-additive solution on 42 and 49 days of storage of red cells Transfusion 1987 27 178 182 10.1046/j.1537-2995.1987.27287150195.x 3824477

[B16] Zimrin AB Hess JR Current issues relating to the transfusion of stored red blood cells Vox Sang 2009 96 93 103 10.1111/j.1423-0410.2008.01117.x 19152602

[B17] Sohmer PR Moore GL Beutler E Peck CC In vivo viability of red blood cells stored in CPDA-2 Transfusion 1982 22 479 484 10.1046/j.1537-2995.1982.22683068607.x 7147326

[B18] Hogman CF Akerblom O Hedlund K Rosen I Wiklund L Red cell suspensions in SAGM medium. Further experience of in vivo survival of red cells, clinical usefulness and plasma-saving effects Vox Sang 1983 45 217 223 10.1111/j.1423-0410.1983.tb01907.x 6414184

[B19] Offner PJ Moore E Biffl WL Johnson J Silliman CC Increased rate of infection associated with transfusion of old blood after severe injury Archives of Surg (Chicago, Ill: 1960) 2002 137 711 716 discussion 716-717. 10.1001/archsurg.137.6.711 12049543

[B20] Koch CG Li L Sessler DI Figueroa P Hoeltge GA Mihaljevic T Blackstone EH Duration of red-cell storage and complications after cardiac surgery N Engl J Med 2008 358 1229 1239 10.1056/NEJMoa070403 18354101

[B21] Weinberg JA McGwin G Jr Griffin RL Huynh VQ Cherry SA Marques MB Reiff DA Kerby JD Rue LW Age of transfused blood: an independent predictor of mortality despite universal leukoreduction J Trauma 2008 65 279 282 discussion 282-274. 10.1097/TA.0b013e31817c9687 18695462

[B22] Watering L Van De Lorinser J Versteegh M Westendord R Brand A Effects of storage time of red blood cell transfusions on the prognosis of coronary artery bypass graft patients Transfusion 2006 46 1712 1718 10.1111/j.1537-2995.2006.00958.x 17002627

[B23] Leal-Noval SR Jara-López I García-Garmendia JL Marín-Niebla A Herruzo-Avilés A Camacho-Laraña P Loscertales J Influence of erythrocyte concentrate storage time on postsurgical morbidity in cardiac surgery patients Anesthesiology 2003 98 815 822 10.1097/00000542-200304000-00005 12657840

[B24] Yap C Lau L Krishnaswamy M Gaskell M Yii M Age of transfused red cells and early outcomes after cardiac surgery Ann Thorac Surg 2008 86 554 559 18640333 10.1016/j.athoracsur.2008.04.040

[B25] Dessertaine G Hammer L Chenais F Rémy J Schwebel C Tabah A Ara-Somohano C Bonadona A Hamidfar-Roy R Barnoud D Timsit JF Does red blood cell storage time still influence ICU survival? Transfusion clinique et biologique: journal de la Société française de transfusion sanguine 2008 15 154 159 10.1016/j.tracli.2008.06.001 18757224

[B26] Kneyber MC Gazendam RP Markhorst DG Plötz FB Length of storage of red blood cells does not affect outcome in critically ill children Intensive Care Med 2009 35 179 180 10.1007/s00134-008-1230-5 18670761

[B27] Pollack MM Patel KM Ruttimann UE PRISM III: an updated Pediatric Risk of Mortality score Crit Care Med 1996 24 743 752 10.1097/00003246-199605000-00004 8706448

[B28] Leteurtre S Martinot A Duhamel A Proulx F Grandbastien B Cotting J Gottesman R Joffe A Pfenninger J Hubert P Lacroix J Leclerc F Validation of the paediatric logistic organ dysfunction (PELOD) score: prospective, observational, multicentre study Lancet 2003 362 192 197 10.1016/S0140-6736(03)13908-6 12885479

[B29] Marshall JC Cook DJ Christou NV Bernard GR Sprung CL Sibbald WJ Multiple organ dysfunction score: a reliable descriptor of a complex clinical outcome Crit Care Med 1995 23 1638 1652 10.1097/00003246-199510000-00007 7587228

[B30] Proulx F Fayon M Farrell CA Lacroix J Gauthier M Epidemiology of sepsis and multiple organ dysfunction syndrome in children Chest 1996 109 1033 1037 10.1378/chest.109.4.1033 8635327

[B31] Leal-Noval SR Muñoz-Gómez M Arellano-Orden V Marín-Caballos A Amaya-Villar R Marín A Puppo-Moreno A Ferrándiz-Millón C Flores-Cordero JM Murillo-Cabezas F Impact of age of transfused blood on cerebral oxygenation in male patients with severe traumatic brain injury Crit Care Med 2008 36 1290 1296 10.1097/CCM.0b013e3181692dfc 18379257

[B32] Weiskopf R Feiner J Hopf H Lieberman J Finlay HE Quah C Kramer JH Bostrom A Toy P Fresh blood and aged stored blood are equally efficacious in immediately reversing anemia-induced brain oxygenation deficits in humans Anesthesiology 2006 104 911 920 10.1097/00000542-200605000-00005 16645441

[B33] Bennett-Guerrero E Veldman TH Doctor A Telen MJ Ortel TL Reid TS Mulherin MA Zhu H Buck RD Califf RM McMahon TJ Evolution of adverse changes in stored RBCs Proc Natl Acad Sci USA 2007 104 17063 17068 10.1073/pnas.0708160104 17940021 PMC2040393

[B34] Reynolds JD Ahearn GS Angelo M Zhang J Cobb F Stamler JS S-nitrosohemoglobin deficiency: a mechanism for loss of physiological activity in banked blood Proc Natl Acad Sci USA 2007 104 17058 17062 10.1073/pnas.0707958104 17940022 PMC2040473

[B35] Kiraly LN Underwood S Differding JA Schreiber MA Transfusion of aged packed red blood cells results in decreased tissue oxygenation in critically injured trauma patients J Trauma 2009 67 29 32 10.1097/TA.0b013e3181af6a8c 19590304

[B36] Relevy H Koshkaryev A Manny N Yedgar S Barshtein G Blood banking-induced alteration of red blood cell flow properties Transfusion 2008 48 136 146 17900281 10.1111/j.1537-2995.2007.01491.x

[B37] Patel MB Proctor KG Majetschak M Extracellular ubiquitin increases in packed red blood cell units during storage J Surg Res 2006 135 226 232 10.1016/j.jss.2006.04.037 16926027

[B38] Lysenko L Mierzchała M Gamian A Durek G Kübler A Kozłowski R Sliwiñski M The effect of packed red blood cell storage on arachidonic acid and advanced glycation end-product formation Arch Immunol Ther Exp (Warsz) 2006 54 357 362 17031463 10.1007/s00005-006-0042-y

[B39] Sweeney J Kouttab N Kurtis J Stored red blood cell supernatant facilitates thrombin generation Transfusion 2009 in press 10.1111/j.1537-2995.2009.02196.x 19413726

[B40] Karam O Tucci M Toledano BJ Robitaille N Cousineau J Thibault L Lacroix J Le Deist F Length of storage and *in vitro *immunomodulation induced by prestorage leukoreduced red blood cells Transfusion 2009 49 2326 2334 10.1111/j.1537-2995.2009.02319.x 19624600

[B41] Zallen G Moore EE Ciesla DJ Brown M Biffl WL Silliman CC Stored red blood cells selectively activate human neutrophils to release IL-8 and secretory PLA2 Shock 2000 13 29 33 10.1097/00024382-200013010-00006 10638666

[B42] Oliver E Carrio ML Rodriguez-Castro D Javierre C Farrero E Torrado H Castells E Ventura JL Relationships among haemoglobin level, packed red cell transfusion and clinical outcomes in patients after cardiac surgery Intensive Care Med 2009 35 1548 1555 10.1007/s00134-009-1526-0 19547956

[B43] Taylor R O'Brien J Trottier S Manganaro L Cytron M Lesko M Arnzen K Cappadoro C Fu M Plisco M Sadaka F Veremakis C Red blood cell transfusions and nosocomial infections in critically ill patients Crit Care Med 2006 34 2302 2308 quiz 2309. 10.1097/01.CCM.0000234034.51040.7F 16849995

[B44] Bilgin YM Watering LM van de Eijsman L Versteegh MI Brand R van Oers MH Brand A Double-blind, randomized controlled trial on the effect of leukocyte-depleted erythrocyte transfusions in cardiac valve surgery Circulation 2004 109 2755 2760 10.1161/01.CIR.0000130162.11925.21 15148271

